# Melanopsin contributions to non-visual and visual function

**DOI:** 10.1016/j.cobeha.2019.06.004

**Published:** 2019-12

**Authors:** Manuel Spitschan

**Affiliations:** 1 Department of Experimental Psychology, University of Oxford, United Kingdom; 2 Centre for Chronobiology, Psychiatric Hospital of the University of Basel (UPK), Switzerland; 3 Transfaculty Research Platform Molecular and Cognitive Neurosciences, University of Basel, Switzerland

## Abstract

Melanopsin is a short-wavelength-sensitive photopigment that was discovered only around 20 years ago. It is expressed in the cell bodies and processes of a subset of retinal ganglion cells in the retina (the intrinsically photosensitive retinal ganglion cells; *ipRGCs*), thereby allowing them to signal light even in the absence of cone and rod input. Many of the fundamental properties of melanopsin signalling in humans for both visual (e.g. detection, discrimination, brightness estimation) and non-visual function (e.g. melatonin suppression, circadian phase shifting) remain to be elucidated. Here, we give an overview of what we know about melanopsin contributions in visual function and non-visual function.

**Current Opinion in Behavioral Sciences** 2019, **30**:67–72

This review comes from a themed issue on **Visual perception**

Edited by **Hannah E Smithson** and **John S Werner**

For a complete overview see the Issue and the Editorial

Available online 28th July 2019

**https://doi.org/10.1016/j.cobeha.2019.06.004**

2352-1546/© 2019 The Author. Published by Elsevier Ltd. This is an open access article under the CC BY license (http://creativecommons.org/licenses/by/4.0/).

## Introduction

Around 20 years ago, the photopigment melanopsin was discovered, first in the skin cells of frogs [1], and subsequently in the retinæ of a wide range of mammals, including humans [2]. In the human retina, melanopsin is expressed in a subset of retinal ganglion cells, rendering them intrinsically photosensitive (intrinsically photosensitive retinal ganglion cells; *ipRGCs*). Only less than 1% of RGCs (<10 000) in the human retina express melanopsin [3]. Signals from these melanopsin-containing cells carry information about light, in addition to the signals arising from cones and rods.

**Figure 1:**
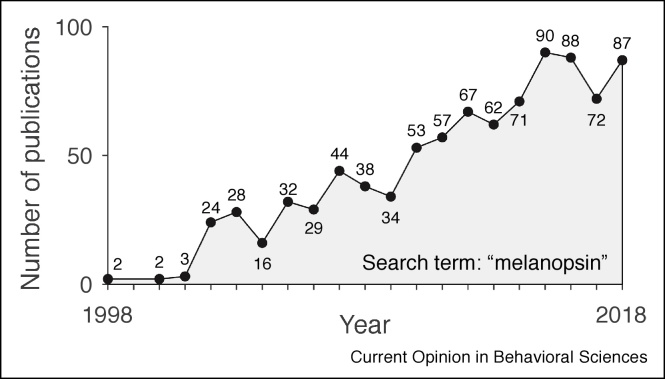
Melanopsin research since 1998. The number of publications incorporating the term ‘melanopsin’ has been steadily increasing since 1998. Frequencies extracted from a PubMed search (27 March 2019).

Since its discovery, the field of study has grown in popularity (Figure 1). At the time of writing this article (mid 2019), research on melanopsin and its contributions is still very much in progress, in particular in humans, where many molecular and genetic techniques used in animal models are not available. The goal of this review is to provide an introduction to what we know about melanopsin function in driving visual (colour and spatial vision) and non-visual function (pupil size regulation, melatonin suppression, circadian photoentrainment).

### Spectral tuning of melanopsin to short-wavelength light

Photopigments such as melanopsin are characterised by their spectral sensitivity, that is, the dependence of their response amplitude to lights of different wavelengths. Generally, the spectral sensitivities of photoreceptors are broad with a distinct unimodal peak at a wavelength to which they are most responsive (*λ*_max_). Photopigments signal light according to the *principle of univariance* [4], which states the output of a photoreceptor (the photocurrent) depends on the total quanta absorbed. This is given by the spectrum of light reaching the receptor weighted by the pigment’s spectral sensitivity. As a consequene, the photoreceptor cannot distinguish between changes in wavelength and changes and intensity.

**Figure 2:**
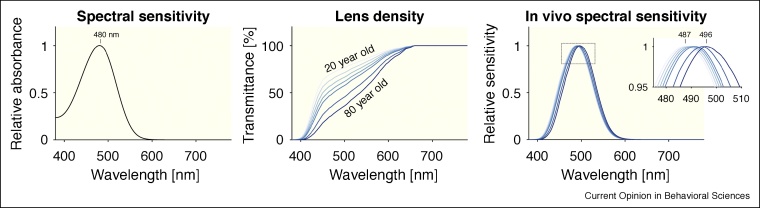
Spectral absorbance of melanopsin (left panel) and pre-receptoral filtering due to lens aging (middle panel). The *in vivo* spectral sensitivity of melanopsin depends on the age of the observer, with peak spectral sensitivities between 487 nm (20-year old observer) and 496 nm (80-year old observer), depending on the age.

Melanopsin absorbs light in the short-wavelength range of the visible spectrum, with *λ*_max_ at or near 480 nm (Figure 2, left panel) [5]. Before light reaches melanopsin expressed in the ipRGCs, however, it passes through the cornea, lens and ocular media. This pre-receptoral filtering alters the spectrum relative to the light arriving at the cornea. The lens specifically attenuates short-wavelength light and increases in density as a function of age (Figure 2, middle panel) [6,7]. Importantly, for melanopsin, the effective *in vivo* spectral sensitivity of melanopsin is shifted from 480 nm to ∼487 nm (for a 20-year-old observer), or ∼496 nm (for an 80-year-old observer).

**Figure 3:**
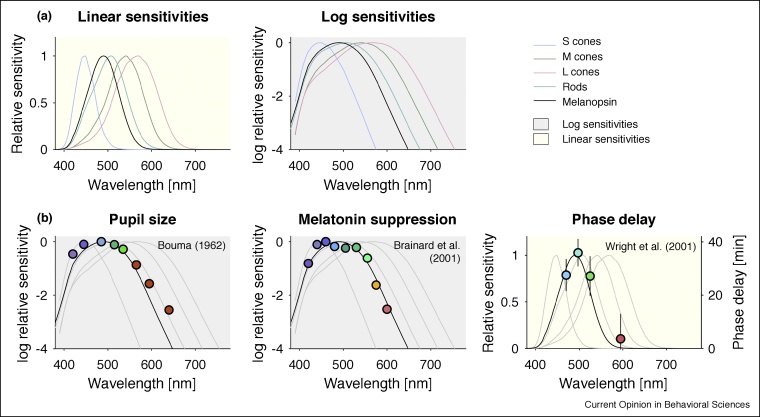
**(a)** Spectral sensitivities are expressed either in linear or logarithmic coordinates. Both are equivalent representations of the spectral sensitivity, but the linear representation ‘squashes’ sensitivity differences at the low end. **(b)** Contributions of melanopsin to pupil size (left panel), melatonin suppression (middle panel), and delaying the circadian clock (right panel). Data were extracted using WebPlotDigitizer. Background color reflects whether shown sensitivities are linear or logarithmic.

Crucially, the spectral sensitivity of melanopsin is distinct from, but heavily overlapping with the spectral sensitivities of the cones and rods (Figure 3a). The principle of univariance and the broad spectral tuning of melanopsin have the consequence that all lights can lead to a melanopsin-encoded signal, if they are bright enough.

An experimental tool to stimulate melanopsin without, in theory, changing visual appearance are *metamers*, which are pairs of spectra which have the property that they are matched in the amount they stimulate cones (and therefore have the same nominal chromaticity and luminance). These two lights may differ in the amount they stimulate melanopsin by a factor which is typically not larger than 3× [8^•^,9^•^,10^•^,^11^,^12^], though this depends on the spectral properties of the primary lights used. Metameric pairs can generated by the method of silent substitution [13].

### Non-visual effects of light mediated by melanopsin

Melanopsin-mediated signals carrying information about light in the environment have a profound influence on our physiology and behaviour. This includes the regulation of pupil size, the acute suppression of melatonin by light, shifting of the phase of our internal clock by light, and the acute modulation of alertness and cardiovascular and thermoregulatory parameters by light (not discussed in this review, see Cajochen [14]).

#### Pupil size regulation

The diameter of the pupil is strongly controlled by melanopsin, in terms of steady-state pupil size [15, 16, 17], the return of pupil size to baseline after light offset [16,18,19], and dynamic pupil responses to, e.g., flicker [11,12,20, 21, 22, 23, 24]. But all photoreceptors contribute to pupil size; to what extent they do depends on the spatial and temporal parameters of the stimulus [25].

#### Melatonin suppression

The production of melatonin, a hormone naturally produced by the body during the evening and night, is suppressed by light [26] via the retinohypothalamic pathway connecting ipRGCs to the suprachiasmatic nucleus (SCN). Two early studies examining the spectral sensitivity of melatonin suppression found tuning inconsistent with cone and rod function [27,28]. Both studies found a peak spectral sensitivity at wavelengths shorter than 480 nm, as one would have predicted from a melanopsin-mediated function. Overall, however, the spectral sensitivity for melatonin suppression is most consistent with melanopsin relative to the other photoreceptors (Figure 3b, middle panel) [29,30]. Importantly, in some functionally blind people with no measurable cone and rod function, light suppresses the production of melatonin [31,32].

#### Circadian phase shifting

Our physiology and behaviour follow a circadian rhythm which is synchronised to the external light–dark cycle via the retinohypothalamic pathway. In turn, exposure to light at night can shift the circadian rhythm by minutes to a few hours. This shift can be either a phase delay or a phase advance, depending on the timing of light exposure (as given by the phase response curve, PRC). Circadian phase shifting is biased towards short wavelength light [33], with evidence for a spectral sensitivity broadly proportional to melanopsin activation (Figure 3b, right panel) [34,35]. Importantly, melatonin suppression and circadian phase are separable and functionally decoupled systems, with neither being a proxy for the other [36^••^,^37^].

#### Other photoreceptor involvement in non-visual functions?

Aside from pupil size, where cone and rod influences have been established, we currently do not have a comprehensive understanding of how cones and rods contribute to the non-visual functions outlined here. In the macaque retina, at least some ipRGC subtypes receive excitatory inputs from L and M cones and rods, and inhibitory input from S cones [38], so cone and rod signals could in principle contribute. In humans, there is some evidence that cones contribute to phase shifting, but that this contribution depends on the timing of the light exposure [39]. Recently, it was also shown that rods may contribute to melatonin suppression [40].

### Melanopsin contributions to vision and visual perception

The possibility that melanopsin signals could contribute to visual function in the classical sense (spatial and colour vision) is tantalising. There is converging evidence that melanopsin signals reach primary visual cortex. ipRGCs in the rodent [41] and primate retina [38] project to dLGN. In humans, pulses of light that only stimulate melanopsin elicit activity in primary visual cortex (V1) as measured with BOLD neuroimaging [11], and this activity cannot be accounted for by inadvertent stimulation of cones. There is mounting psychophysical evidence that melanopsin signals also contribute to detection and discrimination of lights [42,24], brightness estimation [43^•^], and colour perception [24,44].

Demonstrating melanopsin influences to vision is non-trivial, however, and requires very careful methodological scrutiny [45^••^,^46^,^47^]. Using the method of silent substitution [13], melanopsin-stimulating lights can be generated which nominally yield no difference in cone excitation. This experimental approach, however, also faces challenges. For example, cones in the shadow of the retinal blood vessels may be stimulated by stimuli targeted at melanopsin, thereby leading to the inadvertent stimulation of cones [46]. Recently, it was also shown that retinal processing itself, even when the light responses in cones is matched, may introduce inadvertent cone signals [48^•^].

In addition to a direct effect, melanopsin might also influence vision by providing an independent signal for light adaptation, as has been found in rodents [49], thereby modulating cone and rod sensitivity. At present, there is no direct demonstration of such an influence in humans.

#### Melanopsin-mediated spatial vision?

The dendritic field diameter of ipRGCs ranges from ∼250 μm (fovea) to ∼1000 μm (periphery), corresponding to visual angles of ∼0.9° and 3.6°, respectively (ignoring optical factors such blur and chromatic aberration). The receptive fields of the cone inputs, that is, the area of the retina, or of the visual space, over which responses are integrated, are coextensive and relatively large, with receptive field diameters of ∼750 μm [38], corresponding to a visual angle of 2.7° [50]. These receptive fields are rather large compared to the visual resolution for seeing patterns (assuming 1’ = 0.0167° as a conservative estimate for visual resolution), but they *are* finite. With the visual field extending around 150° in the horizontal plane in humans, ipRGCs tile the visual field and are able to provide a spatially selective signal. More concretely, from first principles then, ipRGCs should be able to signal spatial detail. Indeed, the pupil response is also spatially selective [51,52].

Work in rodents found spatial signals carried by melanopsin cells [53], where melanopsin signals can serve as a ‘raumgeber’ [54], akin to the *zeitgebers* (time givers) for circadian synchronisation. Recently, using a novel five-primary display delivering silent-substitution grating stimuli, Allen *et al.* [55^••^] demonstrated that melanopsin may contribute to spatial vision as well, with tuning to low spatial frequencies. To what extent melanopsin helps with visual acuity under natural viewing conditions, however, is at present not known.

## Outlook and conclusion

One of the impediments of arriving at an integrated picture of how rods, cones and melanopsin contribute to retina-mediated effects of light on our perception and our physiology may be that at present, the scientific communities investigating these topics are relatively disparate. The psychophysical enterprise is fundamentally different from the chronobiological enterprise, in terms of the time scales and resources required for experiments and methods used. For example, to assess the amount of phase shift induced by a specific visual stimulus (one condition) requires an in-laboratory protocol of minimum of 34 hours duration under very strictly controlled conditions (e.g. Ref [56]). But the pupil response to the same stimulus class can be characterised within several minutes.

The next decade or so will present great opportunities to integrate vision science, and chronobiology and sleep medicine and synthesize a complete picture from their respective literatures. Tools from vision science, such as the method of silent substitution and metameric lights [13], have begun to find use in chronobiology [8^•^,9^•^,10^•^]. This emerging evidence basis will address the current need of lighting designers, architects, and building engineers to take into account the effect of light on non-visual function in an evidence-based fashion.

## Conflict of interest statement

M.S. has had the following commercial interests in the last two years (2017–18): Investigator-initiated research grants from f.lux Software, BIOS Lighting and Ocean Optics; consulancy contracts with Seoul Semiconductors and Circadian Therapeutics; speaker fees for invited seminars from Seoul Semiconductors and Apple.

## References and recommended reading

Papers of particular interest, published within the period of review, have been highlighted as:

• of special interest
•• of outstanding interest
